# Influence of Artificial Intelligence-Driven Diagnostic Tools on Treatment Decision-Making in Early Childhood Caries: A Systematic Review of Accuracy and Clinical Outcomes

**DOI:** 10.3390/dj11090214

**Published:** 2023-09-12

**Authors:** Abeer Al-Namankany

**Affiliations:** Paediatric Dentistry and Orthodontics Department, College of Dentistry, Taibah University, P.O. Box 41141, Almadinah Almunawwarah 38008, Saudi Arabia; anamankany@taibahu.edu.sa

**Keywords:** dental caries, machine learning, prediction, detection, oral health, systematic review

## Abstract

Early detection and accurate prediction of the risk of early childhood caries (ECC) are essential for effective prevention and management. This systematic review aims to assess the performance and applicability of machine learning algorithms in ECC prediction and detection. A comprehensive search was conducted to identify studies utilizing machine learning algorithms to predict or detect ECC. The included (n = 6) studies demonstrated high accuracy, sensitivity, specificity, and area under the receiver operating characteristic (AUC) values related to predicting and detecting ECC. The application of machine learning algorithms contributed to enhanced clinical decision-making, targeted preventive measures, and improved ECC management. The studies also highlighted the importance of considering multiple factors, including demographic, environmental, and genetic factors, when developing dental caries prediction models. Machine learning algorithms hold significant potential for ECC prediction and detection, having promising performance outcomes. Due to the heterogeneity of the studies, no meta-analysis could be performed. Moreover, further research is needed to explore the feasibility, acceptability, and effectiveness of integrating these algorithms into dental practice. This approach would ultimately contribute to enabling more effective and personalized dental caries management and improved oral health outcomes for diverse populations.

## 1. Introduction

Early childhood caries (ECC) represents a significant public health issue affecting children worldwide. ECC is a term that encompasses any form of caries occurring in infants, toddlers, or pre-schoolers, and it can have severe implications for a child’s overall health, development, and quality of life [1]. The condition often leads to pain, infection, and tooth loss, which can negatively impact a child’s nutrition, speech, and learning abilities [2]. Furthermore, untreated ECC has been linked to higher rates of hospitalization and increased healthcare costs, placing a considerable burden on healthcare systems [3]. As such, accurate and timely diagnosis and treatment are crucial in terms of managing ECC and mitigating its detrimental consequences.

In recent years, the advent of artificial intelligence (AI) has led to considerable advancements in medical diagnostics, including in the field of dentistry. AI-driven diagnostic tools have the potential to improve diagnostic accuracy, facilitate early detection, and support treatment decision-making in a variety of clinical contexts [4]. In particular, AI-powered imaging analysis techniques, such as convolutional neural networks (CNNs) and deep learning algorithms, have shown promising results in terms of detecting carious lesions and assessing their severity [5]. These innovative technologies may offer significant benefits in the management of ECC by enhancing the precision of diagnosis and expediting treatment initiation.

Despite the growing interest in using AI-driven diagnostic tools to identify ECC, the literature on their effectiveness and impacts on clinical outcomes remains limited and heterogeneous in nature. A comprehensive evaluation of the current evidence is warranted to elucidate the roles played by AI technologies in influencing treatment decision-making and improving patient outcomes.

The application of AI in dental diagnostics is an emerging area of study, with several studies demonstrating the potential of these technologies in caries detection and assessment [6,7]. For instance, Lee et al. conducted a study employing a deep learning algorithm to detect and classify carious lesions in dental radiographs [6]. The algorithm demonstrated high levels of accuracy, sensitivity, and specificity, outperforming conventional methods and indicating the potential of AI to act as an effective diagnostic tool [6]. Similarly, Schwendicke et al. explored the use of a CNN to detect occlusal caries in bitewing radiographs, reporting comparable diagnostic performance to those of human examiners and suggesting that AI-based methods could serve as valuable adjuncts to traditional diagnostic techniques [7].

Beyond caries detection, Ngnamsie Njimbouom et al. developed a decision support system based on machine learning algorithms to assist with treatment planning for dental caries [8]. The study revealed that the system significantly improved treatment consistency among dental professionals, highlighting the potential benefits of AI integration into dental practice.

Despite these promising findings, several challenges remain in the translation of AI-driven diagnostic tools into clinical practice. One such challenge is the diversity of the AI algorithms and imaging modalities used in dental diagnostics [9]. This heterogeneity can make it difficult to compare the effectiveness of different AI-driven diagnostic tools and may impede the development of standardized guidelines for their use. Additionally, concerns have been raised about the potential for AI technologies to exacerbate existing health disparities, particularly in terms of access to care and the quality of care provided to underserved populations [10]. As AI-driven diagnostic tools become more prevalent in dental practice, it is essential to ensure that these technologies do not perpetuate or exacerbate existing inequities in oral healthcare.

Another challenge is the need for robust validation of AI algorithms to ensure their generalizability and reliability across different populations and clinical settings [11]. Many AI-driven diagnostic tools have been developed and tested in controlled research environments, and their performances in real-world clinical settings may significantly differ. This issue underscores the importance of validating AI-driven diagnostic tools using diverse and representative datasets, as well as conducting prospective studies to assess their performance and impacts on patient outcomes in actual clinical practice.

Furthermore, the integration of AI-driven diagnostic tools into dental practice raises important ethical and legal considerations, particularly regarding data privacy, informed consent, and professional responsibility [7]. As AI technologies become more integrated into dental practice, it is essential to develop clear guidelines and regulations to address these concerns and ensure the safe and responsible use of AI-driven diagnostic tools.

The successful implementation of AI-driven diagnostic tools in the management of ECC requires effective collaboration between researchers, dental professionals, and other stakeholders, including patients and policymakers [9]. This collaboration is essential to facilitate the translation of research findings into clinical practice, develop appropriate guidelines and regulations, and ensure that AI-driven diagnostic tools are used to improve patient care and outcomes.

Finally, AI has several challenges that remain to be addressed, including the need for robust validation of AI algorithms, addressing ethical and legal considerations, and fostering effective collaboration between stakeholders.

This systematic review aims to synthesize the current evidence regarding the accuracy and clinical outcomes of AI-driven diagnostic tools in ECC management, as well as to provide valuable insights to inform future research, clinical practice, and policy developments.

## 2. Materials and Methods

A comprehensive search was conducted by two independent reviewers to identify studies utilizing machine learning algorithms to predict or detect dental caries. A thorough search of electronic databases, including PubMed, Scopus, Embase, and the Cochrane Library, was performed to identify articles published between 2015 and 2022.

### 2.1. Eligibility Criteria

The inclusion criteria for studies in this systematic review consisted of randomized controlled trials and observational studies published in English, with a minimum of 10 participants per study. These studies should investigate the influence of artificial intelligence-driven diagnostic tools on treatment decision-making in early childhood caries, as well as report the accuracy and clinical outcomes of the tools. Studies with at least a two-week follow-up period were included.

Exclusion criteria for studies encompassed those that did not report on the accuracy and clinical outcomes of artificial intelligence-driven diagnostic tools for early childhood caries, were not published in English, had a follow-up period of less than two weeks, and did not have full-text availability.

### 2.2. Population

The target population for this systematic review comprised children with ECC, including infants, toddlers, and pre-schoolers. Studies that focused on the diagnosis and treatment decision-making of ECC in children from diverse demographic backgrounds and clinical settings were included, ensuring that the results of the review were generalizable and applicable to a wide range of patients.

### 2.3. Intervention and Control

The intervention of interest in this review was the use of AI-driven diagnostic tools for the detection, assessment, and management of ECC. These tools included algorithms based on machine learning, deep learning, and other AI techniques designed to analyze dental images or other clinical data for the purpose of diagnosing and treating ECC. The control group consisted of traditional diagnostic methods, such as visual examination, tactile examination, and radiographic analysis, without the assistance of AI technologies. By comparing the performance of AI-driven diagnostic tools to those of conventional methods, the added value of AI in ECC diagnosis and treatment decision-making was assessed.

### 2.4. Study Type and Size

Randomized controlled trials (RCTs), quasi-experimental studies, and observational studies, such as cohort studies and case-control studies, were included in this review. Both prospective and retrospective studies were considered. To ensure that the included studies provided sufficient evidence of the effectiveness of AI-driven diagnostic tools, only studies with sample sizes of at least 10 participants were included.

### 2.5. Information Sources and Search Strategy

An exhaustive search of electronic databases, such as PubMed, Scopus, Embase, and the Cochrane Library, was performed to find articles published between 2015 and 2022. The detailed database search strategy used is shown in Table 1.

The search strategy incorporated a mix of medical subject headings (MeSH) and free-text terms. Each database had a specific search strategy developed with the help of a medical librarian. The search was finalized in April 2023. The results of the searches were imported into a reference management software program, and duplicate entries were eliminated. Two independent reviewers examined the titles and abstracts for inclusion, and full-text articles that met the eligibility criteria were retrieved. The initial search strategy used is outlined below.

Search terms related to population: “early childhood caries”, “pediatric dental caries”, “child dentistry”, “young children”, “infants”, and “pre-school age children”;

Search terms related to intervention: “artificial intelligence”, “machine learning techniques”, “deep learning approaches”, “diagnostic instruments”, “decision support systems”, and “image analysis”;

Search terms related to outcome: “diagnostic accuracy”, “sensitivity”, “specificity”, “treatment decision-making”, “clinical results”, and “quality of life”.

### 2.6. Study Selection

To identify studies that may be eligible, two separate reviewers screened the titles and abstracts of the articles retrieved. The reviewers then carefully evaluated the full texts of the articles based on pre-determined inclusion and exclusion criteria to determine if they were suitable for inclusion in this study. In the event of any disagreements between the reviewers, they resolved the issue by discussing it or consulting a third reviewer.

### 2.7. Data Collection Process and Data Items

To gather pertinent information from the studies that met the inclusion criteria, a standardized data extraction form was utilized. The two reviewers worked independently during the data extraction process, and any discrepancies were discussed and resolved either between the two reviewers or by involving a third reviewer. The data extraction form included a variety of items, such as the authors, publication year, study design, study setting, population characteristics, sample size, AI algorithms used, diagnostic tools used, accuracy measures, and clinical outcomes.

### 2.8. Summary Measures

The primary outcome measures used in this systematic review were the diagnostic accuracy of AI-driven diagnostic tools compared to conventional methods, as well as the impacts of AI-driven diagnostic tools on treatment decision-making and clinical outcomes in children with ECC. Measures such as sensitivity, specificity, positive predictive value, negative predictive value, and AUC were used to assess diagnostic accuracy. To evaluate the impacts on treatment decision-making and clinical outcomes, measures such as treatment success rates, patient satisfaction, and improvements in oral health-related quality of life were considered.

In this analysis, the extracted data were synthesized using a narrative approach. Potential sources of heterogeneity were explored and, if possible, subgroup analyses were conducted to better understand the influence of various factors on the observed outcomes. Due to the heterogeneity of the studies, no meta-analysis could be performed.

## 3. Results

### 3.1. Study Selection Process

To initiate the selection of studies regarding artificial intelligence techniques, databases were searched, resulting in 1217 citations being retrieved. The first screening step eliminated 814 duplicates, leading to 403 unique titles and abstracts for further scrutiny. The criteria for exclusion were irrelevance (n = 109), population (n = 41), intervention (n = 136), and the type of publication (n = 48), leading to the removal of 334 studies.

After the initial screening, 99 unique full-text studies remained for detailed evaluation. Upon reviewing the full texts, 94 publications were excluded due to population (n = 41), intervention (n = 36), publication type (n = 11), and language (n = 6) issues. Therefore, five studies were deemed to be appropriate for review, with an additional study added through reference list checking, bringing the final number of included studies to six. The systematic review assessed six studies in total.

Liu et al. [12] investigated the creation of a deep learning-based automatic screening system to detect the ectopic eruption of maxillary permanent first molars from panoramic radiographs. They utilized a sample of 1480 patients aged 4 to 9 years old and validated the model using 100 additional panoramic images. The automatic screening system utilized convolutional neural networks (CNNs) and demonstrated positive and negative predictive values of 0.86 and 0.88, respectively, along with a specificity of 0.90 and a sensitivity of 0.86.

Park et al. [13] conducted a study with the goal of developing machine learning-based prediction models for early childhood caries (ECC) and comparing their performance to those of traditional regression models. The study utilized data collected from the Korea National Health and Nutrition Examination Survey (2007–2018), which included 4195 children aged 1 to 5 years old. The researchers developed prediction models using four different algorithms: XGBoost, random forest, LightGBM, and logistic regression. The study found that all four models displayed AUC values ranging between 0.774 and 0.785, demonstrating that the machine learning-based models performed in a manner comparable to that of the traditional logistic regression model in predicting ECC. Interestingly, all four models displayed similar AUC values and misclassification rates, suggesting that machine learning-based models can perform as well as traditional regression models in predicting early childhood caries. This study’s findings are significant as the early detection of dental caries is crucial for prompt treatment and the prevention of further oral health issues. Machine learning-based models could potentially be utilized in clinical settings to provide more accurate predictions and improve the early detection of dental caries.

Karhade et al. [14] aimed to develop and evaluate an automated machine learning algorithm (AutoML) to classify children according to their early childhood caries (ECC) status. The study involved 6404 children aged 3 to 5 years old, who participated in an epidemiologic study of early childhood oral health in North Carolina. The researchers used an AutoML deployment on Google Cloud to evaluate ten sets of ECC predictors to determine classification accuracy, followed by internal validation and external replication. The parsimonious model, which included two terms (children’s age and parent-reported child oral health status), had the highest AUC (0.74), sensitivity (0.67), and positive predictive value (0.64). The performance of the AutoML algorithm in classifying children based on ECC status was comparable to that of the reference model, indicating its potential as a valuable tool for performing ECC screening in young children.

In the study conducted by Ramos-Gomez et al. [15], the researchers explored the use of machine learning algorithms in screening for dental caries in children. The study focused on utilizing parent perceptions of their child’s oral health, which were assessed via survey, as predictors of active caries and caries experience. The sample consisted of 182 parents/caregivers and their children aged 2 to 7 years old living in Los Angeles County, providing a diverse range of participants for analysis. The researchers used the random forest algorithm to identify survey items that were strong predictors of active caries and caries experience. The algorithm identified several significant predictors, including the parent’s age, unmet needs, and the child being African American. These findings emphasize the importance of considering various factors, including demographic factors, when predicting dental caries risk in children. This research demonstrates the potential of machine learning algorithms to perform dental caries screening and highlights the usefulness of algorithm toolkits in helping dental professionals to assess children’s oral health. By utilizing machine learning algorithms, healthcare providers may be able to more effectively identify high-risk groups for dental caries and provide targeted preventive measures.

**Figure 1:**
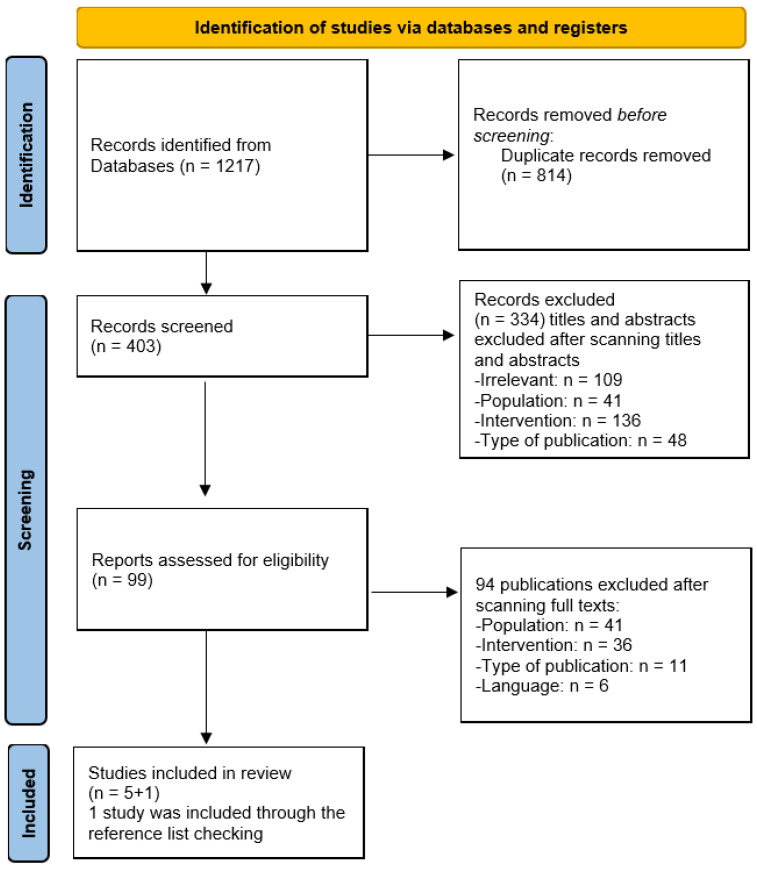
Study selection process.

Lastly, Wu et al. [16] examined the use of machine learning and 16s rRNA sequencing to predict tooth decay by identifying bacterial communities present in an individual’s oral cavity. The study used the oral microbiome of mother–child dyads (both healthy and caries-active samples) in combination with demographic–environmental factors and relevant fungal information to create a multifactorial machine learning model based on the LASSO-penalized logistic regression method. The study identified several bacterial species that were caries predictive, including *Streptococcus mutans*, *Lactobacillus fermentum*, and *Prevotella histicola*. The model demonstrated an AUC of 0.84 for predicting caries in children and 0.87 for predicting caries in mothers, indicating a strong predictive capacity. The researchers found that incorporating demographic and environmental factors into the model led to a slight improvement in caries prediction, emphasizing the importance of considering multiple factors when predicting dental caries risk (Figure 1).

### 3.2. Risk of Bias in Studies

To evaluate the risk of bias in the studies that met the inclusion criteria, appropriate tools were used based on the study design, as indicated in Table 2. For randomized controlled trials (RCTs), the Cochrane Risk of Bias tool [17] was used, while the Quality Assessment of Diagnostic Accuracy Studies-2 (QUADAS-2) tool [18] was employed to perform diagnostic accuracy studies. The risk of bias assessment was conducted independently by two reviewers, and any discrepancies that arose were resolved through discussion or consultation with a third reviewer.

### 3.3. Characteristics of Studies

Table 3 displays the findings of Liu et al. [12], who created a deep learning-based automatic screening system to detect the ectopic eruption (EE) of maxillary permanent first molars (PFMs) using panoramic radiographs. The researchers trained the model on a sample of 1480 patients aged 4–9 years old and tested it on 100 additional panoramic images, achieving positive and negative predictive values of 0.86 and 0.88, respectively, as well as a specificity of 0.90 and a sensitivity of 0.86.

Park et al. [13] aimed to develop machine learning-based prediction models for early childhood caries using data from 4195 children aged 1 to 5 years old. They utilized XGBoost, random forest, LightGBM algorithms, and logistic regression, finding that all four prediction models had AUC values ranging between 0.774 and 0.785.

In Pang et al.’s [19] prospective longitudinal study of 1055 adolescents aged 13 years old, the authors created a caries risk prediction model using a random forest algorithm based on environmental and genetic factors. The model demonstrated an AUC of 0.78 in the training cohort and an AUC of 0.73 in the testing cohort.

Karhade et al. [14] created an automated machine learning algorithm (AutoML) to classify children according to their early childhood caries (ECC) status, using data from 6,404 children aged 3 to 5 years old. The model with the highest AUC, sensitivity, and positive predictive value included two predictors: children’s age and parent-reported child oral health status.

Ramos-Gomez et al. [15] used a machine learning algorithm to screen for dental caries among children based on parent perceptions of their child’s oral health, as assessed via survey. The study found that the parent’s age, unmet needs, and the child being African American were strong predictors of active caries.

Lastly, Wu et al. [16] used machine learning and 16s rRNA sequencing to identify bacterial communities present in an individual’s oral cavity and predict tooth decay. The study’s model had an AUC of 0.84 for predicting caries in children and 0.87 for predicting caries in mothers, incorporating demographic and environmental factors to improve caries prediction. The study identified several caries-predictive bacterial species, including *Streptococcus mutans*, *Lactobacillus fermentum*, and *Prevotella histicola*.

## 4. Discussion

The systematic review analyzed six studies that employed machine learning algorithms in the prediction and detection of dental caries, with each study focusing on different aspects of caries prediction and detection, populations, and interventions. In this study, the key findings were discussed, namely populations, interventions, and other relevant information from each study, while also exploring the similarities, differences, trends, and patterns that emerged.

Liu et al. [12] developed a deep learning-based automatic screening system to detect ectopic eruption of maxillary permanent first molars using panoramic radiographs. The findings suggest that the model developed by Liu et al. has the potential to enhance the clinical diagnosis and management of ectopic eruption by providing a reliable and efficient method of detecting EE in children.

Park et al. [13] aimed to develop machine learning-based prediction models for early childhood caries and compared their performances to those of traditional regression models. This study included a large sample of 4195 children aged 1 to 5 years old, and the researchers created four prediction models using various algorithms: XGBoost, random forest, LightGBM, and logistic regression. The models’ performances were evaluated using AUC values and misclassification rates.

The study’s findings suggest that machine learning algorithms have the potential to effectively identify groups at a high risk of developing early childhood caries. This information can be used to inform targeted preventive measures, such as promoting oral hygiene education and providing dental care to high-risk groups. By leveraging machine learning techniques, it may be possible to create more accurate and reliable ECC risk prediction models, which can be utilized to improve the overall oral health of young children. Moreover, the study highlights the importance of utilizing diverse algorithms to develop prediction models, as the performances of the different models can vary. The use of multiple algorithms can help to ensure that the developed models are robust and accurate, improving the reliability of ECC risk predictions. In summary, Park et al.’s study provides valuable insights into the potential of machine learning algorithms in terms of predicting ECC and highlights the need for continued research in this area.

In a separate study, Pang et al. (2021) constructed a caries risk prediction model for teenagers using a machine learning algorithm that factored in both environmental and genetic factors. The model’s high discrimination ability makes it a potentially valuable tool for identifying high risk of developing caries at the community level. The study’s findings are particularly significant since teenagers form a group that often exhibits high rates of dental caries, and early identification of high-risk individuals can lead to targeted preventive interventions.

This study demonstrates the importance of considering various factors when predicting risk of developing caries in teenagers and highlights the potential of machine learning algorithms in identifying high-risk groups. Overall, the findings of this study could lead to the development of more effective preventive measures to reduce the incidence of dental caries in teenagers.

Karhade et al. [14] aimed to develop and assess an automated machine learning algorithm (AutoML) to classify children based on their early childhood caries status. The researchers utilized Google Cloud’s AutoML deployment to evaluate ten different sets of predictors of ECC and found that the model with only two predictors, namely children’s age and parent-reported child oral health status, had the highest AUC, sensitivity, and positive predictive value. The study’s results suggest that AutoML may be a promising approach for the classification of children based on their ECC status, particularly in resource-limited settings.

Wu et al. [16] investigated the use of machine learning and 16s rRNA sequencing to predict tooth decay by identifying bacterial communities present in an individual’s oral cavity. The study’s multifactorial model demonstrated strong predictive capacity, indicating that considering demographic and environmental factors, in addition to bacterial species, could improve caries prediction accuracy. The study’s results have significant implications for the development of personalized preventive interventions that target bacterial species, as well as demographic and environmental factors associated with an individual’s risk of developing dental caries.

Upon examining the six studies, several similarities and differences emerge. All studies employed machine learning algorithms to perform dental caries prediction or detection, but the specific algorithms and methods used varied. Liu et al. [12] and Karhade et al. [14] focused on utilizing deep learning and AutoML algorithms, respectively, while Park et al. [13], Pang et al. [19], and Ramos-Gomez et al. [15] employed ensemble learning algorithms, such as random forest and gradient boosting. Wu et al. [16] used a LASSO-penalized logistic regression approach.

Another difference between the studies concerns the varied diverse age groups targeted. Liu et al. [12] focused on children aged 4 to 9 years old, Park et al. [13] studied children aged 1 to 5 years old, and Pang et al. [19] targeted teenagers aged 13 years old. Karhade et al. [14] and Ramos-Gomez et al. [15] included children aged 3 to 5 and 2 to 7 years old, respectively, while Wu et al. [16] analyzed mother–child dyads.

The studies also differed in terms of the interventions used and the specific dental caries issues addressed. Liu et al. [12] developed a screening system to detect ectopic eruption, while Park et al. [13], Pang et al. [19], and Karhade et al. [14] focused on predicting early childhood caries and the risk of developing caries in children and teenagers. Ramos-Gomez et al. [15] aimed to identify survey items used to predict dental caries in children, and Wu et al. [16] explored the potential use of the oral microbiome to predict tooth decay.

Despite these differences, all studies demonstrated the potential of machine learning algorithms in dental caries prediction and detection, with most models showing high accuracy, sensitivity, specificity, and AUC values. The studies highlighted the importance of considering multiple factors, such as demographic, environmental, and genetic factors, when predicting dental caries risk. Furthermore, the studies emphasized the potential benefits of using machine learning algorithms to implement targeted preventive measures and improved clinical decision-making in dental caries management.

In comparing the outcomes of the individual studies, it is evident that the machine learning models generally achieved satisfactory predictive performances. Liu et al.’s [12] automatic screening system for detecting ectopic eruption had high sensitivity and specificity, while the machine learning-based prediction models for early childhood caries in Park et al.’s [13] study displayed AUC values comparable to those of traditional regression models. Pang et al.’s [19] caries risk prediction model for teenagers demonstrated high discrimination ability, and Karhade et al.’s [14] AutoML algorithm performed comparably to the reference model in terms of classifying children based on their ECC status. Ramos-Gomez et al.’s [15] random forest algorithm successfully identified survey items as predictors of active caries, and Wu et al.’s [16] LASSO-penalized logistic regression model had strong capacity to predict caries in both children and mothers.

The populations studied in these research projects varied not only in terms of age, but also other demographic factors. For instance, Ramos-Gomez et al. [15] found that certain demographic factors, such as the child being African American, played a significant role in predicting active caries. This issue highlights the importance of considering demographic factors when developing dental caries prediction models, as these factors may influence the accuracy and generalizability of the models.

In terms of interventions, the studies employed different machine learning algorithms to address specific dental caries issues. Some studies focused on early detection of dental issues, such as Liu et al. [12], who developed a screening system for ectopic eruption. Other authors, such as Park et al. [13] and Pang et al. [19], aimed to predict early childhood caries or caries risk. The interventions used in these studies showcase the versatility of machine learning algorithms in addressing various dental caries-related issues.

In summary, the six studies analyzed in this systematic review highlight the potential of machine learning algorithms to predict and detect ECC across different populations and interventions. Despite the differences in the algorithms used, populations studied, and interventions employed, the studies collectively demonstrate the effectiveness of machine learning algorithms in dental caries prediction and detection.

The models generally achieved high accuracy, sensitivity, specificity, and AUC values, emphasizing their potential in informing targeted preventive measures and improving clinical decision-making in dental caries management. Furthermore, these studies underscore the importance of considering multiple factors, such as demographic, environmental, and genetic factors, when developing dental caries prediction models.

Ngnamsie Njimbouom et al. developed a decision support system based on machine learning algorithms to assist in treatment planning for dental caries [8]. A similar study of the use of AI was also reported on the regarding the detection of fluoride concentration in drinking water in Turkey, and the results showed that the use of AI was cheaper, faster, and more feasible than the use of many chemical analysis techniques available in the laboratory [20].

Despite the promising findings of this systematic review, there are several limitations that should be acknowledged. Firstly, the number of studies included in this review is relatively small, as most researchers tend to avoid dealing with children. As machine learning applications in dental caries prediction and detection is an emerging area of research, the limited number of studies may not comprehensively cover all aspects of this field.

Secondly, the populations studied in these research projects vary not only in terms of age, but also other demographic factors. This issue may limit the generalizability of the findings, as the performances of machine learning models may differ across diverse populations with different demographic characteristics.

Thirdly, the studies included in this review employed different machine learning algorithms, which could affect the comparability of their results. The variations in algorithms used and the specific dental caries-related issues addressed make it challenging to draw definitive conclusions regarding the optimal machine learning algorithm for dental caries prediction and detection.

Lastly, the studies analyzed in this review primarily focused on the predictive performances of machine learning models. While these models demonstrated high accuracy, sensitivity, specificity, and AUC values, the implementation of these models in real-world clinical settings and their impacts on patient outcomes require further investigation. Future studies should continue to explore the potential of machine learning algorithms in dental caries prediction and detection, with a focus on improving the generalizability and applicability of these models in diverse populations and settings. Furthermore, they should explore the feasibility, acceptability, and effectiveness of integrating machine learning algorithms into dental practice to better understand their potential benefits and challenges.

## 5. Conclusions

The systematic review and analysis of the six studies demonstrate the potential of artificial intelligence algorithms to predict and detect ECC across different populations and interventions. Due to the heterogeneity of the studies, no meta-analysis could be performed. The findings reveal that machine learning algorithms can be a promising way to enhance the prediction, detection, and management of ECC by achieving high accuracy, sensitivity, specificity, and AUC values. It seems that the versatility of these algorithms can allow targeted preventive measures, improved clinical decision-making, and tailored interventions for ECC management. Moreover, these studies emphasize the importance of considering multiple factors, such as demographic, environmental, and genetic factors, when developing dental caries prediction models. This comprehensive approach can better inform the development of ECC prediction models, contributing to their generalizability and applicability in diverse populations and settings. However, further research is needed to explore the feasibility, acceptability, and effectiveness of integrating these algorithms into dental practice. This approach would ultimately contribute to more effective and personalized dental caries management and improved oral health outcomes for diverse populations.

## Figures and Tables

**Table 1:** Search strategy.

Database	Search Terms
PubMed	(“early childhood caries” [MeSH terms] or “early childhood caries” [All fields] or “pediatric dental caries” [MeSH terms] or “pediatric dental caries” [All fields] or “child dentistry” [MeSH terms] or “child dentistry” [All fields] or “young children” [MeSH terms] or “young children” [All fields] or “infants” [MeSH terms] or “infants” [All fields] or “pre-school age children” [MeSH terms] or “pre-school age children” [All fields]) and (“artificial intelligence” [MeSH terms] or “artificial intelligence” [All fields] or “machine learning techniques” [MeSH terms] or “machine learning techniques” [All fields] or “deep learning approaches” [MeSH terms] or “deep learning approaches” [All fields] or “diagnostic instruments” [MeSH terms] or “diagnostic instruments” [All fields] or “decision support systems” [MeSH terms] or “decision support systems” [All fields] or “image analysis” [MeSH terms] or “image analysis” [All fields]) and (“2015/01/01” [PDAT] to “2022/12/31” [PDAT])
Scopus	TITLE-ABS-KEY (“early childhood caries” or “pediatric dental caries” or “child dentistry” or “young children” or “infants” or “pre-school age children”) and TITLE-ABS-KEY (“artificial intelligence” or “machine learning techniques” or “deep learning approaches” or “diagnostic instruments” or “decision support systems” or “image analysis”) and PUBYEAR >2014 and PUBYEAR <2023 and (LIMIT-TO (DOCTYPE, “ar”))
Embase	(“early childhood caries”/exp or “early childhood caries” or “pediatric dental caries”/exp or “pediatric dental caries” or “child dentistry”/exp or “child dentistry” or “young children”/exp or “young children” or “infants”/exp or “infants” or “pre-school age children”/exp or “pre-school age children”) and (“artificial intelligence”/exp or “artificial intelligence” or “machine learning techniques”/exp or “machine learning techniques” or “deep learning approaches”/exp or “deep learning approaches” or “diagnostic instruments”/exp or “diagnostic instruments” or “decision support systems”/exp or “decision support systems” or “image analysis”/exp or “image analysis”) and ([embase]/lim not ([embase]/lim and [medline]/lim) and (2015:2022)
The Cochrane Library	((“early childhood caries”) or (“pediatric dental caries”) or (“child dentistry”) or (“young children”) or (“infants”) or (“pre-school age children”)) and ((“artificial intelligence”) or (“machine learning techniques”) or (“deep learning approaches”) or (“diagnostic instruments”) or (“decision support systems”) or (“image analysis”)) and (Publication date >2014 and Publication date <2023)
Google Scholar	(“early childhood caries” or “pediatric dental caries” or “child dentistry” or “young children” or “infants” or “pre-school age children”) and (“artificial intelligence” or “machine learning techniques” or “deep learning approaches” or “diagnostic instruments” or “decision support systems” or “image analysis”) and (after 2014/12/31 and before 2023/01/01)
ProQuest Dissertation and Thesis	(AB (“early childhood caries”) or AB (“pediatric dental caries”) or AB (“child dentistry”) or AB (“young children”) or AB (“infants”) or AB (“pre-school age children”)) and (AB (“artificial intelligence”) or AB (“machine learning techniques”) or AB (“deep learning approaches”) or AB (“diagnostic instruments”) or AB (“decision support systems”) or AB (“image analysis”)) and PD (2015–2022)

**Table 2:** Results of risk assessment.

Study	Risk of Bias				Applicability Concerns		
	Patient Selection	Index Test	Reference Standard	Flow and Timing	Patient Selection	Index Test	Reference Standard
Liu et al. [12]	Low risk	Unclear	Low risk	Low risk	Low risk	Low risk	Low risk
Wu et al. [13]	Low risk	Low risk	Low risk	Unclear	Low risk	Low risk	Low risk
Park et al. [14]	Low risk	Low risk	Unclear	Low risk	Low risk	Low risk	Low risk
Pang et al. [15]	Low risk	Low risk	Low risk	Low risk	Low risk	Low risk	Low risk
Karhade et al. [16]	Unclear	Low risk	Low risk	Low risk	Low risk	Low risk	Low risk
Ramos-Gomez et al. [17]	Low risk	Low risk	Low risk	Low risk	Low risk	Low risk	Low risk

Based on the risk analysis, no studies were excluded.

**Table 3:** Characteristics of studies.

	Author	Year	Study Type	Algorithm	Objective	Outcome	Author’s Observation
1	Liu et al. [12]	2022	Cross-sectional	CNNs	Develop a semi-automatic model to detect ectopic eruption of maxillary first molars in 4–9-year-olds’ radiographs	High sensitivity and specificity in automated screening	The algorithm may enhance clinical diagnosis and management of ectopic eruption
2	Wu et al. [16]	2021	Cross-sectional	ANNs	Create an ML model to identify caries-related oral microbes in mother–child dyads	Desirable results for both mothers and children	Further refinement needed by considering more variables
3	Park et al. [13]	2021	Cross-sectional	ANNs	Predict early childhood caries using ML-based AI models (XGBoost, random forest, and Light GBM algorithms)	Favorable performance in dental caries prediction with satisfactory AUC values	Helpful in identifying high-risk groups and applying preventive measures
4	Pang et al. [19]	2021	Cross-sectional	ANNs	Develop a caries risk prediction model for teenagers by considering environmental and genetic factors	Accurate identification of individuals at high and very high risk of developing caries	Potential as a powerful tool for performing community-level high caries risk identification
5	Karhade et al. [14]	2021	Cross-sectional	ANNs	Evaluate the accuracy of an automated ML algorithm for early childhood caries (ECC) classification	Comparable performance to that of the reference model (AUC: 0.74, sensitivity: 0.67, PPV: 0.64)	Valuable tool for ECC screening
6	Ramos-Gomez et al. [15]	2021	Cross-sectional	ANNs	Identify survey items to predict dental caries in children using a machine learning algorithm	Algorithm toolkits can help dental professionals to assess children’s oral health	Demonstrates potential for dental caries screening in children

## Data Availability

Not applicable.
